# Chondromyxoid fibroma of the temporal bone: A rare case report

**DOI:** 10.1097/MD.0000000000019487

**Published:** 2020-03-13

**Authors:** Tao Liu, Jing Yao, Xiaoyu Li, Xinmeng Qi, Pengyun Zhao, Zhiqiao Tan, Jie Wang, Yongxin Li

**Affiliations:** a Department of Otorhinolaryngology, Affiliated Hospital of Jining Medical University; b Department of Pharmacology, Jining Medical University, Jining; c Department of Otorhinolaryngology, Beijing Tongren Hospital; d Department of Otolaryngology, Beijing Shuangqiao Hospital, Beijing, the People's Republic of China.

**Keywords:** diagnosis, temporal bone cartilage myxoidfibroma, treatment

## Abstract

**Rationale:**

Chondromyxoid fibroma (CMF) is a rare form of benign bone tumor and easily misdiagnosed as fibrosarcoma. Hence, to explore the clinical manifestations, diagnostic tests, and therapeutic procedures for temporal bone cartilage myxoid fibroma, it is important to optimize patient treatment and avoid overtreatment. Previous research has discussed cases of CMF, but this paper presents a systematic, complete, and comprehensive introduction of this disease based on this case and related literature.

**Patient concerns:**

A 52-year-old male patient presented with pain in his right ear for 2 years and hearing loss in his right ear with tinnitus for 1 year. The patient had a history of hypertension for 9 years and it was well-controlled.

**Diagnosis:**

A computed tomography (CT) scan of the temporal bone showed an expansive growth on the right temporal bone plate and tympanic plate, presenting as a cloud-like ground glass opaque shadow involving the temporom and ibular joint, middle skull base, and small auditory bones. A magnetic resonance imaging (MRI) of the temporal bone showed a large and irregular soft tissue mass shadow on the right temporal bone plate. The right temporal bone plate was occupied by the lesion, consistent with a bone origin. From the results of the imaging examination of the patient, a lesion occupying the temporal bone in the right ear and mastoiditis in the right middle ear was initially diagnosed.

**Interventions:**

Right ear temporal bone tumor resection and abdominal fat extraction were conducted.

**Outcomes:**

Postoperative pathological results demonstrated myxoid fibroma of the temporal bone cartilage. No recurrence or severe complications were observed in 8 months of follow-up.

**Lessons:**

A finding of myxoid fibroma of the temporal bone cartilage is rare in the clinic. The growth of such tumors is slow. The temporal bone CT and inner ear MRI were helpful in diagnosis. Surgery was the principal treatment.

## 1 Introduction

Chondromyxoid fibroma (CMF) is a rare benign bone tumor, first proposed and described in detail by Jaffe and Lichtenstein in 1948.^[1]^ It can easily be misdiagnosed as fibrosarcoma and accounts for approximately 0.5% of all bone tumors, 2% of the benign bone tumors, 71% of the cases affecting the long bones of the lower limbs on the epiphyseal aspect, most commonly in the knee joint (accounting for 40%), and 8% in the foot bones.^[2,3]^ Only 2% of CMFs occur in the skull or facial bones,^[2]^ most frequently presenting in the maxilla or mandible. From a review of the literature, CMF in the temporal bone is extremely rare.^[4,5]^ Moreover, the onset of CMF is insidious and the course of the disease is relatively long, lasting for years or even decades. Depending on the location within the temporal bone, presentation of these lesions is usually subtle and accompanied by numerous symptoms, previously described as headache, hearing loss, facial pain, facial nerve paralysis, otalgia, tinnitus, and diplopia due to the compression and involvement of neural structures.^[4]^ However, systemic symptoms are not apparent and are often ignored. In this paper, a detailed and comprehensive review of the clinical diagnosis and treatment of 1 case of temporal bone CMF, including general data, auxiliary examination, scanning, imaging, and surgical treatment, were combined with the related literature to analyze and summarize the clinical manifestations, imaging characteristics, and treatment strategies of the disease, in order to improve the early diagnosis and timely treatment of such diseases.

## 2 Case presentation

The patient was a 52-year-old man who presented with right ear pain for 2 years and hearing loss with tinnitus for 1 year. There was no apparent cause for the patient's right ear needle-like pain, which reduced after approximately10 seconds but recurred repeatedly. Moisture with no smell and no purulent secretion exuded from the right ear. Headache, fever or facial paralysis, dizziness, nausea or vomiting did not occur, but the patient's jaw opening was restricted. The patient had been diagnosed with otitis externa by the local hospital but his symptoms had not improved following drug treatment 2 years previously. The patient began to suffer hearing loss in his right ear, accompanied by tinnitus and cicada chirping 1 year ago. The pain expanded to the temporal region, with intermittent onset, accompanied by a reduced range of motion in his jaw combined with pain. Physical examination by a specialist found no deformity in the bilateral auricles, a moist right ear canal with downward distension of the wall, and no abnormal infection but perforation of the eardrum. The left ear canal was unobstructed with the tympanic membrane complete.

**Figure 1:**
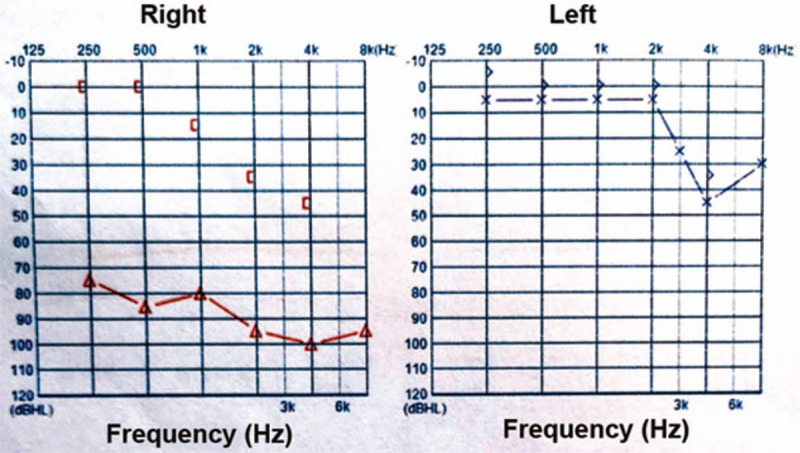
Results of pure tone audiometry of the CMF patient. (A) Bone conduction: 0-15-35-45 dB HL; air conduction: 88-80-98-100 dB HL (right ear); (B) bone conduction: 0-0-0-35 dB HL; air conduction: 5-5-5-45 dB HL (left ear). CMF = chondromyxoid fibroma.

**Figure 2:**
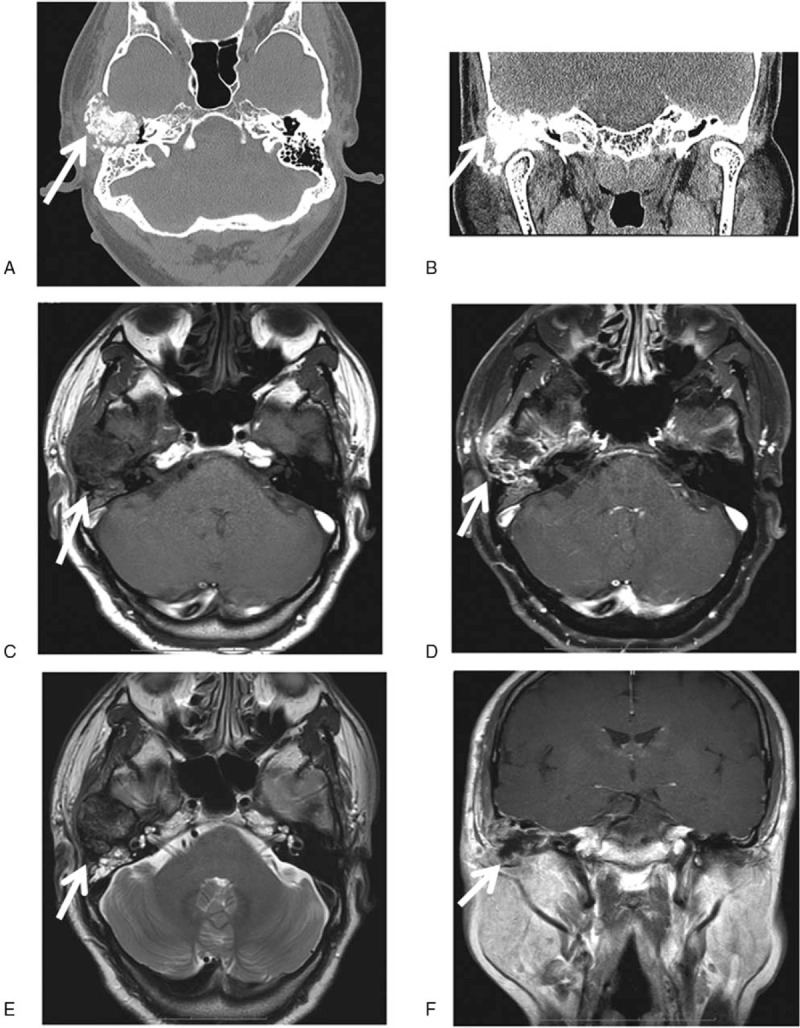
Temporal bone CT scan and MRI results of the CMF patient: (A) CT axial plane images, (B) CT coronal scan. The results display the right temporal bone plate and tympanic plate presenting with swelling, cloudy flocculent ground-glass opacity, clear boundary, thin bone in the middle of the cranial fossa, low continuity, and involving the temporom and ibular joint. The lesion exhibited hypointensity and presented as an oval soft tissue shadow on the axial T1-weighted image (C, arrow) and T1-weighted image of the coronal aspect (F, arrow). Heterogeneous hyperintensity on the axial T2-weighted image (E, arrow) and T1-enhanced weighted image of the axial position shows ring enhancement (D, arrow). CMF = chondromyxoid fibroma, CT = computed tomography, MRI = magnetic resonance imaging.

Pure tone audiometry (Fig. 1) indicated bone conduction with 0-15-35-45 dB HL and air conduction with 88-80-98-100 dB HL in the right ear and bone conduction with 0-0-0-35 dB HL and air conduction with 5-5-5-45 dB HL in the left ear. A CT scan of the temporal bone (Fig. 2A and B) found: an expansive growth on the right temporal bone plate and tympanic plate, presenting as a cloud-like ground glass opaque shadow involving the temporom and ibular joint, middle skull base, and small auditory bones. It was initially considered to be an osteogenic lesion with osteoblastoma and an additional magnetic resonance imaging (MRI) was recommended. Inflammation in the right external auditory meatus suggested that middle ear mastoiditis was more likely, with high bilateral jugular fossa and low left cranial fossa. MRI of the temporal bone (Fig. 2C–F) found a large and irregular soft tissue mass shadow on the right temporal bone plate, showing long T1 and mixed short T2 signals, appearing to be annular after enhancement, with multiple small septa around the periphery. The lesion had pushed the zygomatic process forward, the mastoid process backward, and the tympanum inward. The right temporal bone plate was occupied by the lesion, consistent with a bone origin. The diagnosis on admission was lesion occupying the right ear temporal bone and right middle ear mastoiditis.

Right ear temporal bone tumor resection and abdominal fat extraction were conducted under general anesthesia. After the subcutaneous injection of adrenal brine into the right retroauricular region, a “C”-shaped incision was created, extending forward to the front of the tragus. The skin and subcutaneous tissue were separated from the temporalis muscle layer and a musculoskeletal flap with a pedicle was constructed in front of the ear. The external auditory canal was removed along the surface plane of the mastoid process and the skin of the ear canal trimmed and closed by folding outwards, to strengthen the perichondrium.

**Figure 3:**
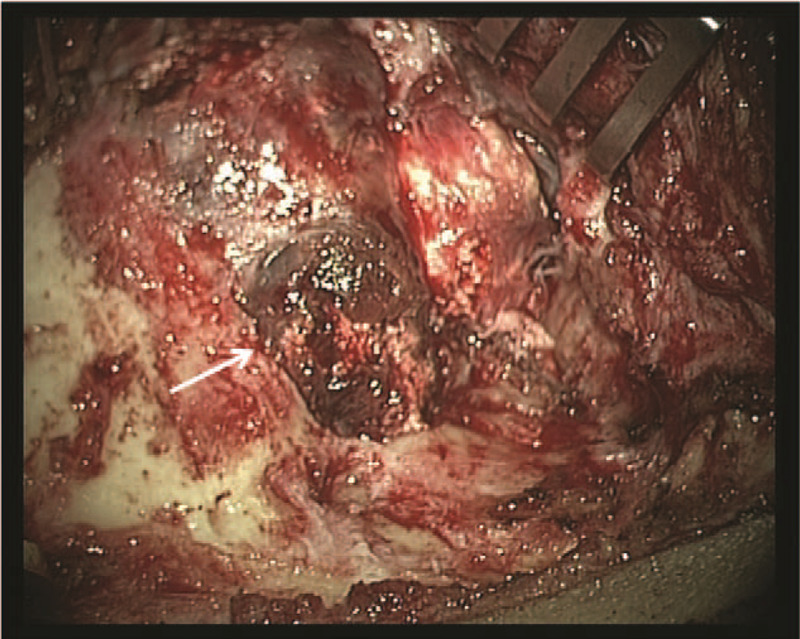
Operative picture of the CMF patient. Anterior and superior region of the mastoid process, the posterior and anterior wall of the external auditory canal, and the root of the posterior zygomatic arch were destroyed by the tumor. The mass was bulbous and expanded, protruding forward into the posterior, internal, and lateral m and ibular joint. CMF = chondromyxoid fibroma.

After exposure of the mastoid region, it was apparent that the anterior and superior region of the mastoid process, the posterior and anterior wall of the external auditory canal, and the posterior root of the zygomatic arch had been destroyed by the tumor. The mass was bulbous and expansive, protruding forward into the posterior, internal, and lateral mandibular joint (Fig. 3). Bone was gradually removed along the periphery of the tumor to fully expose the meningeal horn of the sinus.

**Figure 4:**
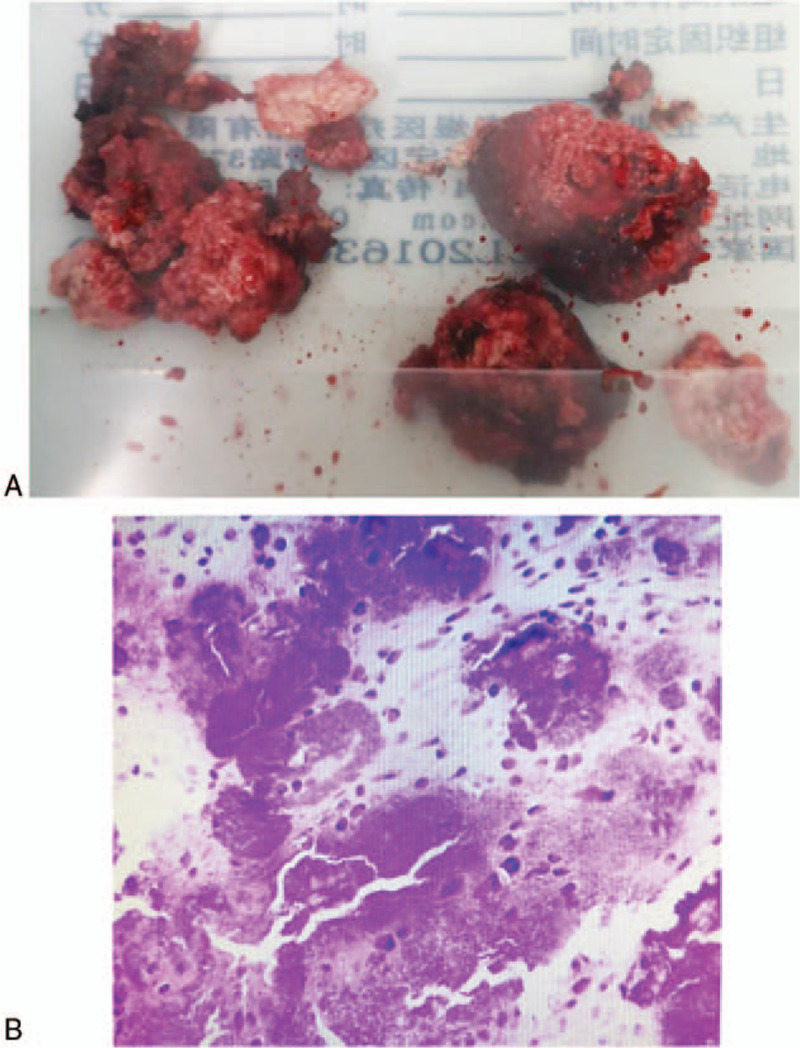
Surgical resection specimen and histopathological results of the CMF patient. (A) Yellowish-white gravel-like bone inside the tumor, with no apparent blood supply, with a small dark gray liquefaction region in the center. (B) The chondrogenic tumor (right ear temporal bone) with clear, granular, calcification, and hemorrhage, which was considered benign. Diffuse calcification and hemorrhage were also found and it was difficult to define the type from morphological and immunohistochemical staining results that did not exclude chondromyxoid fibroma. CMF = chondromyxoid fibroma.

Resection and volume reduction were conducted in the middle of the tumor, which had a yellowish-white gritty interior with no apparent blood supply and a small dark gray region of liquefaction at the center (Fig. 4A). The mastoid cavity was sufficiently delineated and the damaged posterior wall of the external auditory canal further trimmed down to its lower level. The remaining skin and eardrum of the external auditory canal were cleaned and the incus and stapes bones separated. Then, the malleus and incus were removed together with the mass.

After examination of the anterior wall of the external auditory canal, the bone defect could be seen in this tissue, with the mass surrounding the temporom and ibular joint. After the tumor was carefully cleaned from the lateral aspect of the temporom and ibular joint capsule, it was found to be intact. Separation along the superior, anterior, and posterior boundaries of the mass demonstrated that it was located within the middle cranial fossa. The mass was strongly attached to the dura mater of the middle cranial fossa and difficult to separate. After removal of the tumor, the middle cranial fossa meningeal membrane exhibited a cribriform pattern with 3 punctate damaged regions. Following fixation of a strip of temporalis muscle with silk thread, no apparent cerebrospinal fluid outflow was observed. The lumen was irrigated and no residual tumor was found.

The temporalis muscle was used for sealing the tympanostomy of the eustachian tube, with bone wax used for reinforcement. Abdominal fat transplantation was performed, the incision at the posterior auricle sutured in a contralateral position, and the wound dressed with pressure bandages.

**Figure 5:**
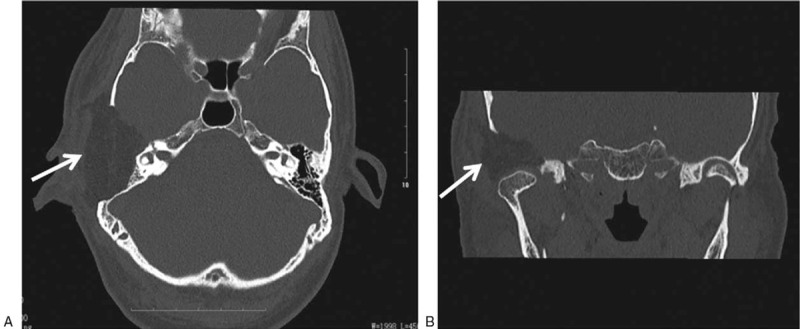
Temporal bone CT of the CMF patient after surgery. (A) The transverse section and (B) the coronal section. CMF = chondromyxoid fibroma, CT = computed tomography.

Postoperative histopathological analysis of paraffin sections of the right temporal bone found a chondrogenic tumor accompanied by clear granular calcification, hemorrhage, and tissue cell reaction, which was considered benign. Although the type of tumor was difficult to define, combined with histomorphology and immunohistochemical staining results, CMF was not excluded. Immunohistochemistry demonstrated that the cells in the mass were CD68(+), CK(–), Ki-67 index (approximately 5%), S-100(+), and CD1α(+) (Fig. 4B). The patient recovered well after surgery, without facial nerve paralysis, difficulty opening his mouth, cerebrospinal fluid otorrhea, fever, headache, nausea, or vomiting. A postoperative CT examination, as shown in Fig. 5A and B, demonstrated postoperative changes to the temporal bone with no new biological residue found. The stitches were removed 10 days after surgery and the patient was discharged after recovery.

No recurrence or severe complications were observed in 8 months of follow-up.

## 3 Discussion

CMF is a benign bone tumor originating from cartilage tissue, which is more commonly seen in the metaphysis of long bones.^[6–8]^ Only 1% of the reported cases of CMF have occurred in the head or neck and it is especially uncommon in the skull base, especially within the temporal bone.^[9]^ A summary of the literature describing CMF in the head and neck over the past decade, in which 29 cases were found, are listed in Table 1. This exemplifies the unusual nature of CMF in the temporal bone and that the systemic symptoms are not typical, demonstrating that such a tumor can be easily missed and the patient misdiagnosed.

**Table 1:**
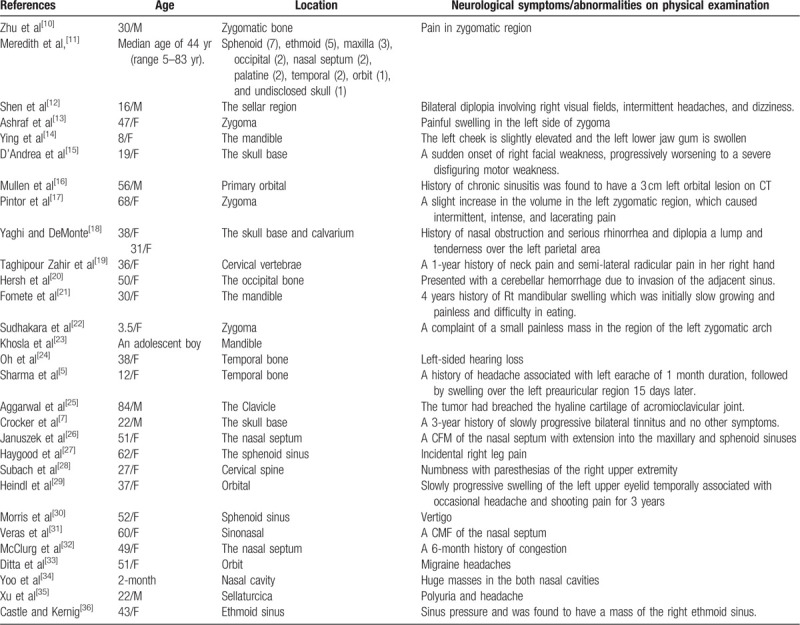
Summary of published cases of chondromyxoid fibroma in the head and neck during 2009 to 2019.

CMF is characterized by lobules of spindle-shaped or stellate cells with abundant myxoid or chondroid intercellular material with varying numbers of multinucleated giant cells of different sizes.^[37]^ Histopathologic analysis of CMF reveals a myxoid lesion with a center containing few cells surrounded by stromal cells and reactive boney spicules within the hyaline cartilage with up to 75% of the lesions in the skull and facial bones also containing calcified matrix.^[8,31]^

The imaging features of the temporal bone cartilage myxoid fibroma in this case were typical. CT and MRI are useful modalities for preoperative diagnosis and were able to indicate the boundary of the temporal osteochondralmyxoid fibroma and the relationship between the tumor and surrounding structure. The imaging characteristics of CMFs in temporal bone have been described in detail.^[5,6,8,24,38–40]^ X-ray images demonstrate a radioopaque lesion with a clear border.^[41]^ CT imaging can effectively indicate osteolytic lesions with marginal sclerosis^[2,42]^ and reveal the structure of the bone around the lesion. Giant cell tumors on the temporal bone are mostly clearly defined in CT images, with margins that are sclerotic and fan-shaped.^[6]^ Intracellular calcification has been reported in up to 75% of the CMFs of the skull base,^[4]^ in which case, intra-tumor calcification was visible, not only on CT images but also in histology (Fig. 4). MRI is more effective in determining the extent of disease than CT. CMF tissue exhibits a low density on T1-weighted imaging (Fig. 2C) and non-uniform high intensity on T2-weighted imaging (Fig. 2E). T2 signal inhomogeneity is caused by changes in chondroid, myxoid, and fibrous components in the lesion. In addition, temporal bone CMFs usually present with gadolinium enhancement, as shown in Fig. 2D. The results shown in Fig. 2C–F are consistent with the characteristics previously described.

In temporal bone lesions, it is important to distinguish CMFs from other tumors, including chordoma, myxoidchondrosarcoma, and facial schwannoma. Chordomas usually occur in the middle of the skull base and extend outward to the bone,^[24]^ which can be easily distinguished from giant cell tumors using immunohistochemistry. Chordomas usually express epithelial antigens, such as those of the epithelial membrane and keratin. CMFs are not stained by antibodies against these proteins.^[43]^ It is important to distinguish between CMF and myxoidchondrosarcomas because their treatments are different. They have similar MRI features and so are difficult to differentiate and both are positive for S-100 in immunohistochemical analysis.^[39,44]^

Since CMF may be misdiagnosed as chondrosarcoma with poor prognosis and required more radical surgery, the appropriate diagnosis of this benign entity is crucial.^[45,46]^ CT is the diagnostic basis for chondrosarcomas, which are most common at the base of the skull in the head and neck and rarely occur in other bones, such as the maxilla, mandible, or laryngeal cartilage. Imaging results reveal that chondrosarcomas are destructive lobulated masses that also exhibit regions of calcification that tend to be in the form of spots or curved and annular shapes rather than being amorphous. Morphologically, they contain hyaline cartilage and are often associated with myxoid changes. Chondrocytes are atypical with enlarged nuclei that are often detailed. The immunophenotype of chondrosarcomas is typically positive for vimentin and S-100 but without the expression of epithelial markers, such as keratin or EMA. Chondrosarcomas are malignant tumors with a 5-year survival rate of 40% to 80%. They usually require radical resection to obtain negative margins, whereas other therapies have little effect.^[47]^

CMF is a benign chondrogenic tumor that should be treated by surgical resection. Local resection or simple curettage of the affected bone in the treatment of CMF is feasible, although there is a tendency for recurrence after simple curettage at a rate of 12.5% to 25%, with an interval as long as 30 years. Therefore, extensive focal or total resection of the affected bone can reduce recurrence and is the most appropriate treatment.^[48]^

In summary, we report a case of temporal bone CMF in this study, a rare tumor. There was no systemic or local recurrence. As CMF and chondrosarcoma have some similarities in imaging and morphology, CMF often lacks specificity in clinical manifestation and is easily missed or misdiagnosed. Clinically, the tumor grows slowly, with the patient is generally not conscious of any obvious symptoms, which may occasionally be observed in x-ray examination. When there are symptoms, the most common manifestations are local chronic pain, swelling, limited mobility, and rare pathological fractures.^[49]^ The present case occurred in the temporal bone plate and the principal presentation was pain and discomfort around the ear without other systemic symptoms. Early symptoms of temporal bone cartilage myxoid fibroma lack specificity and are easy to misdiagnose. Detailed investigation of the medical history, comprehensive physical examination of the patient, and corresponding auxiliary examination may reduce misdiagnosis as temporal bone cartilage mucoid fibroma, but prompt surgical excision will result in effective treatment. This summary may allow clinics to optimize their treatment and avoid overtreatment of CMF.

## Author contributions

**Data curation:** Xinmeng Qi, Pengyun Zhao.

**Project administration:** Jie Wang, Yongxin Li.

**Writing – original draft:** Tao Liu, Jing Yao.

**Writing – review & editing:** Tao Liu, Xiaoyu Li, Zhiqiao Tan.
